# The role of probiotics in preventing dental caries: a systematic review of clinical evidence

**DOI:** 10.3389/froh.2025.1720036

**Published:** 2025-12-02

**Authors:** Alessio Danilo Inchingolo, Angelo Michele Inchingolo, Irene Palumbo, Mariafrancesca Guglielmo, Lilla Riccaldo, Roberta Morolla, Francesco Inchingolo, Andrea Palermo, Gianna Dipalma

**Affiliations:** 1 Department of Interdisciplinary Medicine, University of Bari “Aldo Moro”, Bari, Italy; 2 Department of Biomedical, Surgical and Dental Sciences, Milan University, Milan, Italy; 3 Department of Experimental Medicine, University of Salento, Lecce, Italy

**Keywords:** probiotics, prevention, dental caries, *s.mutans*, *lactobacillus*, *bifidobacterium*, oral probiotics

## Abstract

**Background:**

Probiotics have emerged as a promising adjunctive strategy for oral health, particularly in the prevention of dental caries, a multifactorial disease driven by ecological imbalances in the oral microbiome.

**Methods:**

A systematic search of PubMed, Scopus, and Web of Science was conducted for studies published between January 2014 and January 2025, focusing on the use of probiotics for caries prevention in children and young adults. Clinical trials and observational studies were included, and a qualitative synthesis was performed based on the extracted outcomes.

**Results:**

Twenty-one studies met the inclusion criteria. Most clinical trials reported a significant reduction in *Streptococcus mutans* levels following administration of probiotic strains, particularly *Lactobacillus paracasei, Lactobacillus rhamnosus, and Bifidobacterium lactis*. Several studies also demonstrated a decreased incidence of new carious lesions and an improvement in salivary immune markers. However, a minority of studies found no significant effect, highlighting potential variability due to strain type, dosage, delivery method, and population characteristics.

**Conclusion:**

The current evidence supports the potential of specific probiotic strains to reduce cariogenic bacterial loads and contribute to caries prevention. Further standardized, long-term trials are needed to clarify the most effective formulations and regimens for clinical application.

**Systematic Review Registration:**

PROSPERO CRD42025646287.

## 1 Introduction

Dental caries is a chronic disease with a multifactorial etiology and represents one of the major challenges in global oral health (1, 2). According to the World Health Organization (WHO), approximately 2.3 billion people worldwide are affected by caries in permanent teeth, while over 530 million children suffer from caries in deciduous teeth (3, 4). This condition not only compromises oral functionality and aesthetics but also has a significant impact on quality of life, nutritional status, and psychological well-being (5, 6). Traditionally regarded as an infectious and transmissible disease, dental caries is now understood to be the result of microbial dysbiosis within the dental biofilm, favored by environmental, behavioral, and dietary factors (7, 8).

The oral biofilm is a complex and dynamic microbial community that colonizes dental surfaces in a stratified and organized manner (9–11). Under conditions of eubiosis, the oral microbiota plays an active role in host defense, modulating immune responses and preventing colonization by exogenous pathogens (12, 11, 13). However, increased intake of fermentable sugars, poor oral hygiene, and changes in salivary composition can lead to an ecological shift toward a more acidogenic and aciduric microbiota, thereby promoting enamel demineralization and the onset of carious lesions (14–16). In this context, bacterial species such as *Streptococcus mutans, Lactobacillus spp., Scardovia wiggsiae, and Actinomyces spp*. have been widely recognized as key etiological agents in caries progression (17–19).

In recent years, scientific interest has increasingly focused on the use of biological strategies to restore microbial balance within the oral biofilm, among which the use of probiotics has emerged as a promising approach (20–22). Probiotics are defined by the Food and Agriculture Organization (FAO) and the World Health Organization (WHO) as “live microorganisms which, when administered in adequate amounts, confer a health benefit on the host.” Originally developed in the context of gastrointestinal health, the concept of probiotics has been progressively extended to the oral cavity, giving rise to the term “oral probiotics,” referring to bacterial strains capable of positively modulating the oral microbiota and preventing or alleviating conditions such as caries, periodontal diseases, halitosis, and mucositis (22–24).

Probiotics exert their beneficial effects through multiple mechanisms, including: competition for adhesion sites on dental surfaces, production of antimicrobial metabolites (bacteriocins, organic acids, hydrogen peroxide), modulation of salivary pH, inhibition of growth and metabolic activity of oral pathogens, stimulation of local immune defenses, and reinforcement of mucosal barrier function (25–27). Furthermore, emerging evidence suggests that probiotics may influence host inflammatory responses by interacting with toll-like receptors (TLRs) and regulating cytokine production (28–30).

Various bacterial strains have been studied for their probiotic potential in the oral environment (11, 31, 32). Among the most investigated are *lactobacilli*, particularly *Lactobacillus rhamnosus GG, L. reuteri, L. casei Shirota,* and *L. paracasei*, which are known for their ability to produce bacteriocins and inhibit the growth of *S. mutans* (33–35). However, the role of lactobacilli remains controversial, as some species (such as *L. fermentum* and *L. acidophilus*) have been associated with caries progression. Therefore, strain selection is a critical factor in the development of effective and safe oral probiotics (36–38).

Other strains of interest include bifidobacteria (*Bifidobacterium lactis, B. bifidum*), which can interfere with pathogen adhesion and stimulate mucosal immune responses, and oral streptococci such as *Streptococcus salivarius K12* and *M18*. The latter, in particular, is known for its ability to produce lantibiotics (salivaricin A and B), which selectively inhibit *S. mutans*, and has shown potential in reducing dental plaque and carious lesions in preliminary clinical studies (39–41). Recently, less conventional strains such as *Weissella cibaria*—which produces hydrogen peroxide and reduces coaggregation between *Fusobacterium nucleatum* and *S. mutans*—and commensal colonizers like *Streptococcus oralis* and *S. dentisani* have also been investigated for their anticariogenic properties (42–44).

The delivery methods for oral probiotics are diverse and include orally disintegrating tablets, chewing gums, mouthwashes, lozenges, yogurt, fermented milk, and sublingual sprays (45–47). The efficacy of probiotic interventions depends not only on the selected strain but also on the delivery vehicle, microbial viability, dosing frequency, and duration of administration. The ability of probiotic strains to adhere to and persist in the oral cavity is a key determinant of sustained clinical benefits, though colonization is often transient (48–50).

Scientific evidence supporting the use of probiotics for the treatment and prevention of carious lesions has expanded considerably in recent years, with an increasing number of controlled clinical trials, meta-analyses, and systematic reviews (51–53). However, the findings remain heterogeneous and sometimes conflicting, due to variability in study designs, intervention durations, target populations, and outcome measures (e.g., bacterial load, salivary pH, incidence of new lesions) (54–56). Some studies have demonstrated significant reductions in S. mutans counts and plaque accumulation following probiotic administration, while others have reported no clinically meaningful changes. Additionally, it remains to be clarified whether probiotics can serve not only in primary prevention but also as adjuvants in the management of incipient non-cavitated carious lesions, potentially promoting enamel remineralization in synergy with fluoride and other agents (57–59).

The rising prevalence of dental caries among pediatric patients, orthodontic wearers, individuals with hyposalivation, and persons with disabilities underscores the urgency of developing complementary preventive strategies alongside conventional protocols. If validated by robust scientific evidence, oral probiotics could represent a non-invasive, accessible, and well-tolerated option for modulating the biofilm and preventing dental caries. However, further well-designed clinical trials with long-term follow-up are needed to confirm the efficacy and safety of specific probiotic strains, evaluate their interactions with other therapeutic agents, and determine their capacity for stable colonization of the oral cavity (60–62).

In recent years, numerous studies have explored the use of probiotics in oral health; however, the evidence regarding their specific preventive role against dental caries remains fragmented and inconsistent. The present systematic review aims to provide a comprehensive and updated synthesis of clinical research published over the past decade, focusing on the efficacy of different probiotic strains, delivery systems, and dosages in reducing cariogenic bacterial load and preventing lesion development. Particular attention is given to pediatric and young adult populations, who are most susceptible to early microbial dysbiosis, and to the immunological mechanisms underlying probiotic action, such as modulation of salivary antimicrobial peptides. By critically evaluating the quality and methodological robustness of available studies, this review seeks to clarify current uncertainties and offer evidence-based insights to guide future preventive strategies in dentistry.

In light of these considerations, the present systematic review aims to critically analyze the current evidence regarding the use of probiotics in the prevention and management of carious lesions, assessing the effectiveness of different bacterial strains, delivery methods, target populations, and clinical outcomes. The objective is to provide an updated and rigorous overview to guide clinical practice and stimulate future research in the evolving field of oral microbiotherapy.

## 2 Materials and methods

### 2.1 Protocol and registration

The protocol was registered at PROSPERO with the ID CRD42025646287, and the systematic review was carried out in accordance with the Preferred Reporting Items for Systematic Reviews and Meta-Analyses (PRISMA) guidelines.

### 2.2 Search processing

Papers were found related to probiotics and their potential role in the prevention of dental caries and the maintenance of oral health by searching PubMed, Scopus, and Web of Science between 1 January 2014 and 31 January 2025. (“Dental Caries” OR “Caries” OR “Tooth Decay”) AND (“Probiotics” OR “Probiotic”) were the Boolean keywords utilized in the search strategy (Table 1). The search was limited to publications from January 2014 onward to capture the most recent decade of clinical evidence, reflecting advances in probiotic formulations and contemporary diagnostic criteria for dental caries.

**Table 1:** Descriptive summary of item selection.

Author (Year)	Country	Population (Age, *n*)	Probiotic strain(s)	Delivery method & duration	Primary outcome(s)	Secondary outcome(s)	Main findings/Effect direction
Duraisamy et al. 2021 (76)	India	40 children (6–12 years)	*Lactobacillus spp*.	Probiotic/synbiotic curd, 15 days	S. mutans CFU/mL	Salivary pH	↓ *S. mutans* in both groups; probiotic > synbiotic
Sarmento et al. 2019	Brazil	40 children	*L. casei*	Cheese fortified with probiotics, 28 days	Oral microbiota composition	Acceptability	↓ potentially cariogenic flora; good compliance
Janiani et al. 2022 (74)	India	34 children (3–6 years)	*L. rhamnosus*	Probiotic milk, 1 week	Salivary S. mutans	Plaque score	↓ *S. mutans* short-term; no long-term effect
Invernici et al. 2020	Brazil	30 adults (periodontitis)	*Bifidobacterium animalis subsp. lactis HN019*	Lozenges, 30 days	Clinical periodontal indices	Salivary immune markers	Improved PD, BOP; ↑ salivary IgA
Lee et al. 2021	Korea	100 adults	*Weissella cibaria CMU*	Tablets, 8 weeks	Halitosis score	QoL	↓ volatile sulfur compounds; improved oral comfort
Staszczyk et al. 2022 (82)	Poland	140 children (3–6 years)	*L. salivarius (heat-inactivated)*	Chewable tablets, 2 weeks + 1-year follow-up	Caries incidence	Plaque index	↓ caries increment; stable plaque index
Laleman et al. 2020	Belgium	39 adults (periodontitis)	*L. reuteri ATCC PTA 5289* *+* *DSM 17938*	Lozenges/drops, 12 weeks	Pocket depth	Plaque, BOP	↓ PPD ≥ 1 mm at 24 weeks; ↓ BOP
Santana et al. 2022	Brazil	36 edentulous adults	*Multi-strain (L. rhamnosus, L. paracasei, B. lactis)*	Oral gel + capsules, 12 weeks	Peri-implant mucositis (BOP)	IL-1β, TNF-α	↓ BOP; ↓ inflammatory cytokines
Laleman et al. 2019	Belgium	10 adults (peri-implantitis)	*L. reuteri ATCC PTA 5289* *+* *DSM 17938*	Lozenges + drops, 12 weeks	PPD change	Plaque, BOP	↓ PPD, ↓ BOP; no microbiological difference
Schlagenhauf et al. 2020	Germany	72 navy sailors	*L. reuteri DSM 17938* *+* *ATCC PTA 5289*	Lozenges, 42 days	Gingival index	Plaque index	↓ GI and PI in probiotic group
Kang et al. 2020	Korea	92 adults (20–39 years)	*Weissella cibaria CMU*	Tablets, 8 weeks	BOP, PI	Oral microbiota	↓ BOP; ↓ F. nucleatum, *S. aureus*
Hasslöf et al. 2022 (72)	Sweden	38 preschoolers	*L. reuteri DSM 17938* *+* *ATCC PTA 5289*	Drops, 12 months	Caries recurrence	S. mutans	↔ no significant difference; high attrition
Teanpaisan et al. 2024 (83)	Thailand	60 children (7–10 years)	*L. paracasei SD1*	Milk drink, 6 months	S. mutans CFU/mL	HNP1–3, caries increment	↓ *S. mutans*; ↑ HNP1–3; slower caries progression
Pørksen et al. 2023 (80)	Denmark	52 children (4–6 years)	*L. rhamnosus GG*	Milk drink, 8 weeks	S. mutans levels	Salivary pH	↓ *S. mutans*; ↑ pH; short-term effect only
Uyar et al. 2024 (73)	Turkey	45 children	*L. rhamnosus SP1*	Milk supplement, 10 weeks	Caries incidence	β-defensin-3	↓ DMFT; ↑ β-defensin-3; moderate correlation
Mughal et al. 2024 (70)	Pakistan	80 schoolchildren	*L. acidophilus* *+* *B. bifidum*	Yogurt, 6 weeks	S. mutans reduction	Plaque index	↓ S. mutans, ↓ plaque index
Costa et al. 2023	Brazil	64 children	*L. rhamnosus GG* *+* *L. casei 431®*	Cheese, 6 months	Caries increment	Salivary buffering	↓ DMFT; ↑ buffering capacity
Sharma et al. 2022	India	50 children	*L. brevis CD2*	Lozenges, 15 days	Oral mucositis (chemo patients)	Plaque score	↓ mucositis severity; ↓ plaque
Wang et al. 2023	China	90 children	*L. plantarum*	Milk, 3 months	S. mutans	Salivary pH	↓ *S. mutans*; ↑ pH
Saini et al. 2024	India	70 children (6–9 years)	*L. rhamnosus GG*	Milk, 4 weeks	S. mutans CFU/mL	Plaque index	↓ *S. mutans*, ↓ plaque; transient
Peltola et al. 2015	Finland	60 children	*B. lactis BB-12*	Yogurt, 6 months	S. mutans levels	DMFT	↓ *S. mutans*; no DMFT difference

↑ = increase; ↓ = decrease; ↔ = no significant change.

CFU, colony forming units; DMFT/dmft, decayed, missing, and filled teeth; ICDAS, International Caries Detection and Assessment System; PPD, probing pocket depth; BOP, bleeding on probing; QoL, quality of life; HNP, human neutrophil peptide.

### 2.3 Inclusion criteria

The following were the inclusion criteria: (1) studies that examined the probiotics and their potential role in the prevention of dental caries and the maintenance of oral health; (2) randomized clinical trials, retrospective studies, case–control studies, case series, case report and prospective studies; (3) English language; and (4) full-text studies. Papers that did not match the above criteria were excluded.

The review was conducted using the following PICOS criteria:

Participants: children and young adults. For the purposes of this review, “children and young adults” were considered to include individuals approximately between the ages of 2 and 25 years. In cases where broader age ranges were reported (e.g., 18–60 years), studies were included only if data specific to the younger subgroup could be extracted, or if the sample was predominantly composed of individuals within the defined age range.

Interventions: administration of probiotics through any formulation (e.g., milk, lozenges, curd, drops, mouth rinses) with the purpose of modifying oral microbiota composition or affecting dental caries development.use of different probiotics for preventing caries.

Comparisons: control groups receiving placebo or standard diet/oral care without probiotic supplementation.no use of probiotics in the diet.

Outcomes: probiotics were investigated for their effects on dental caries incidence and on oral microbial, biochemical, or immunological parameters.

Study design: Only randomized controlled trials (RCTs) published in English were included. Non-randomized, retrospective, *in vitro*, or animal studies were excluded.

### 2.4 Exclusion criteria

Non-randomized clinical studies, retrospective analyses, case series, case reports, and narrative reviews were excluded to ensure methodological consistency and minimize risk of bias.

### 2.5 Data processing

Three reviewers (M.G., I.P., and R.M.) independently searched the databases to find the studies and evaluated their quality based on the selection criteria. The selected articles were downloaded using version 6.0.15 (Corporation for Digital Scholarship, Vienna, VA, USA). To settle any disputes between the three authors, a senior reviewer (F.I.) was consulted. A meta-analysis was not performed due to the substantial heterogeneity among the included studies in terms of probiotic strains, dosage regimens, delivery formats, outcome measures, and follow-up durations, which made quantitative synthesis inappropriate and potentially misleading.

### 2.6 Quality assessment

The methodological quality of all included RCTs was assessed using the revised Cochrane Risk of Bias tool (RoB 2) (63). Three reviewers (M.G., I.P., and R.M.) independently performed the assessment. Each of the five points under evaluation had a bias level assigned to it. A third reviewer (F.I.) was consulted in cases of disagreement until an agreement was reached. Among the domains evaluated by ROBINS were the following:

• D1—Randomization process
• ⁠D2—Deviations from the intended interventions
• ⁠D3—Missing outcome data
• ⁠D4—Measurement of the outcome
• ⁠D5—Selection of the reported result

## 3 Results

### 3.1 Study selection

A total of 1,604 publications (Scopus *N* = 780, PubMed *N* = 373, and Web of Science *N* = 451) were found using the electronic database search; no papers were found through the manual search.

**Figure 1:**
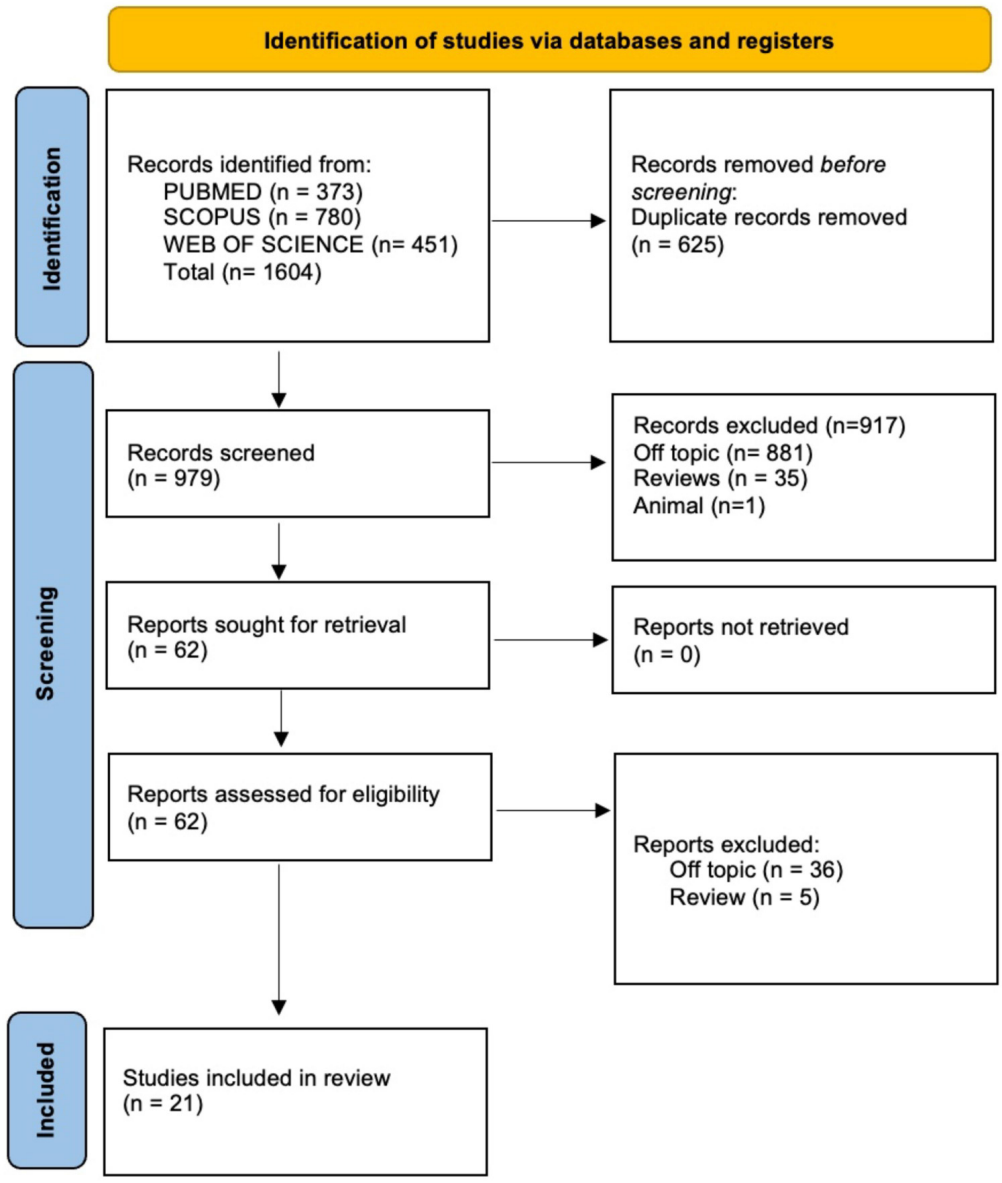
Literature search following the preferred reporting items for systematic reviews and meta-analyses (PRISMA) flow diagram and database search indicators.

Following the removal of duplicates (*N* = 625), the abstracts and titles of 979 studies were assessed for screening. Of these, 63 records were chosen for review after 917 papers (881 off-topic, 35 reviews, and 1 animal study) failed to meet the inclusion requirements. Of these, 42 reports (36 off-topic, 6 reviews) were eliminated because they did not fit the inclusion criteria. 21 records were chosen for qualitative study after being deemed eligible. Figure 1 depicts the selection procedure, and Table 1 provides a description of the records that were selected. A total of 21 randomized controlled trials were included in the qualitative synthesis. Among these, 15 studies (71.4%) reported a statistically significant reduction in salivary *Streptococcus mutans* counts, 9 studies (42.8%) documented a decrease in caries incidence or progression, and 6 studies (28.6%) described improvements in salivary immune markers, including defensins and neutrophil peptides. Conversely, 4 studies (19%) found no significant effect of probiotic administration on cariogenic parameters.

The heterogeneity among studies mainly concerned the probiotic strains (predominantly *Lactobacillus paracasei, L. rhamnosus, Bifidobacterium lactis*), delivery vehicles (milk, lozenges, yogurt, mouthrinses), and treatment duration (ranging from 24 days to 12 months). Despite methodological differences, the majority of studies consistently showed a trend toward microbial modulation and short-term caries reduction following probiotic supplementation.

### 3.2 Outcome

#### 3.2.1 Microbiological outcomes

Most included trials demonstrated a decrease in cariogenic bacterial counts, primarily *Streptococcus mutans*, following probiotic consumption. Studies using *Lactobacillus paracasei SD1, L. rhamnosus SP1, and Bifidobacterium lactis Bb12* consistently reported reductions in SM levels and improved salivary buffering capacity.

#### 3.2.2 Clinical outcomes

Several trials evaluated the incidence or progression of dental caries. A significant reduction in new or cavitated lesions was reported in studies using probiotic-enriched milk or lozenges, while a few trials found no statistically significant effect. Variations in strain type, dosage, and follow-up length may explain these discrepancies.

#### 3.2.3 Immunological outcomes

A subset of studies explored immune-related parameters, showing increased levels of salivary antimicrobial peptides such as human neutrophil peptides (HNP1–3) and *β*-defensin-3 after probiotic intake. These findings suggest that probiotics may enhance local immune defense mechanisms in addition to microbiological modulation.

### 3.3 Summary of findings

Across all included studies, the majority showed favorable microbiological outcomes, while fewer demonstrated clear clinical improvements or immunological effects.

The collective evidence suggests that probiotics can alter oral microbiota composition and support caries prevention, particularly in pediatric and adolescent populations. However, the heterogeneity of probiotic strains, delivery systems, treatment durations, and outcome measures limits direct comparison and meta-analytic synthesis.

Future studies should adopt standardized caries assessment methods (DMFT/dmft or ICDAS), include long-term follow-up, and integrate both microbiological and immune biomarkers to elucidate mechanisms of action and the durability of probiotic effects.

### 3.4 Methodological features

Several studies have evaluated the role of probiotic dairy products, particularly probiotic milk and yogurt, in reducing cariogenic bacterial load and preventing caries in children.

Rodríguez et al. and Sandoval et al. investigated *L. rhamnosus* SP1-enriched milk in high-risk preschool children, reporting a significant reduction in caries progression compared to standard milk (64, 65). Similarly, Yadav et al. found that probiotic milk containing *L. casei* Shirota significantly reduced salivary SM levels (66).

Studies by Manmontri et al. and Wattanarat et al. on *L. paracasei* SD1 demonstrated reduced SM counts and enhanced salivary immune markers in children with severe early childhood caries (ECC) (67, 68). Likewise, Javid et al. found that probiotic yogurt containing B. *lactis* Bb12 significantly decreased S.M. and lactobacilli levels in students with initial-stage caries (69).

These findings suggest that probiotic dairy products can beneficially modulate the oral microbiome and reduce cariogenic bacterial populations.

Probiotic lozenges and drops have been explored as adjuncts to conventional oral care. Pørksen et al. and Mughal assessed probiotic-prebiotic lozenges (*Lacticaseibacillus rhamnosus*, *L. paracasei*, and 2% arginine) in children using fluoride toothpaste, showing trends toward reduced caries progression, though statistical significance was inconsistent (70, 71).

Hasslöf et al. examined *Limosilactobacillus reuteri* drops for ECC prevention but found no significant impact on caries recurrence, likely due to study limitations (72). Sakaryalı Uyar et al. evaluated *Bifidobacterium*-based probiotic drops in children post-full-mouth dental rehabilitation, observing a temporary reduction in SM levels, though the effect diminished over time (73).

Probiotic curd has been investigated for its ability to reduce cariogenic bacterial load.

Duraisamy et al. and Sakhare et al. found that probiotic curd (L. *acidophilus*, *B. lactis*) significantly reduced SM levels in schoolchildren, with superior effects compared to synbiotic curd. Janiani & Ravindran compared probiotic milk (Yakult) and probiotic powder (enKor-D), both demonstrating significant reductions in SM counts (74–76).

Probiotic mouth rinses have been compared to traditional antimicrobial agents.

Krupa et al. found that probiotic rinses were as effective as xylitol in reducing SM levels by 28% in children (77). Ghasemi et al. reported that probiotic yogurt and xylitol gum significantly reduced SM counts, though effects diminished over time (78).

Gizani et al. investigated *L. reuteri* in orthodontic patients but found no significant reduction in WSL, highlighting the need for further research in this high-risk group.

Probiotics may influence oral immune responses (79).

Wattanarat et al. and Manmontri et al. observed increased salivary neutrophil peptides (HNP1-3) following probiotic consumption, suggesting enhanced immune defense and reduced cariogenic bacterial levels (67, 68).

In addition to the widespread reduction in Streptococcus mutans levels, the included studies reported mixed outcomes regarding the impact of probiotics on caries progression. While several trials observed a decrease in the incidence of new carious lesions, others did not find statistically significant differences compared to control groups. This variability may be attributed to differences in probiotic strains, dosage, delivery methods, treatment duration, and population characteristics. Furthermore, some studies indicated a potential immunomodulatory effect, as reflected by increased levels of salivary antimicrobial peptides following probiotic administration. However, the beneficial effects appeared to be transient in many cases, with bacterial levels often returning to baseline shortly after discontinuation of the intervention. Taken together, these findings suggest that while most probiotic formulations exhibit short-term benefits in reducing cariogenic bacterial load and modulating salivary immune factors, the clinical relevance and long-term sustainability of these effects remain to be further elucidated, as discussed in the following section.

### 3.5 Quality assessment and risk of bias of included articles

**Figure 2:**
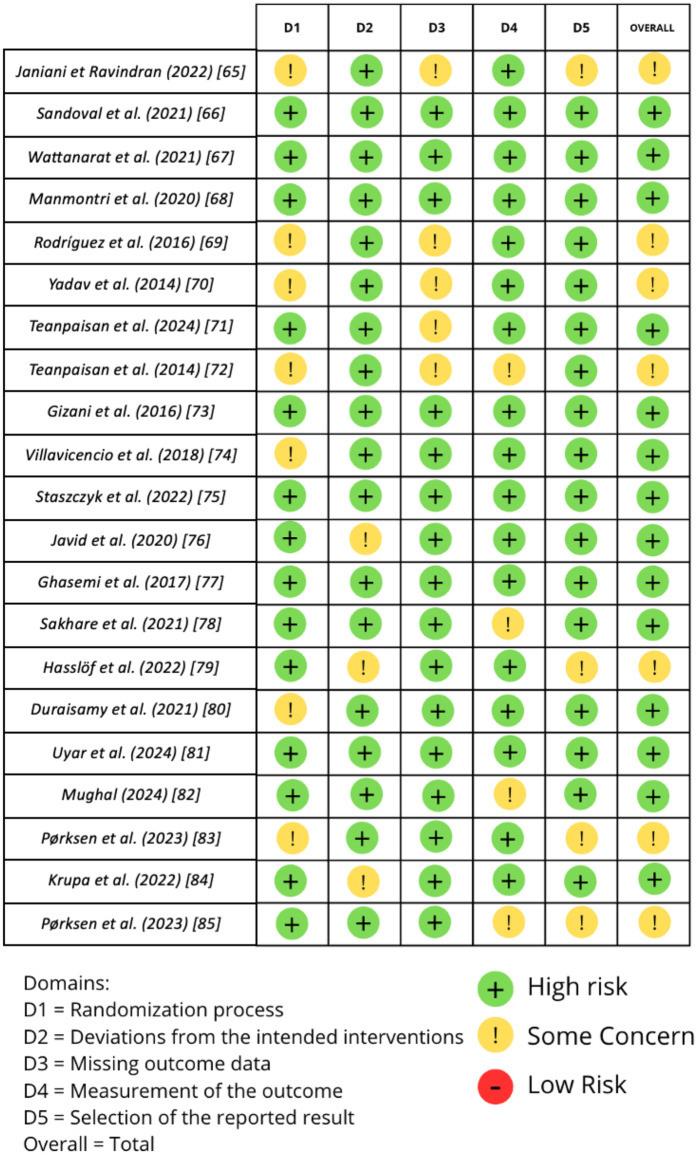
Bias assessment. The figure highlights the proportion of studies categorized as having low, high, or unclear risk of bias in each domain, providing a visual summary of the overall methodological quality of the included studies.

The methodological quality of the 21 randomized controlled trials (RCTs) included in this review was assessed using the revised Cochrane Risk of Bias tool (RoB 2) across five domains: randomization process (D1), deviations from intended interventions (D2), missing outcome data (D3), measurement of outcomes (D4), and selection of reported results (D5). Overall, most studies were judged as presenting a low to moderate risk of bias. Trials with larger sample sizes, longer follow-up, and robust blinding procedures—such as the multicenter studies by Wattanarat et al., Manmontri et al., Villavicencio et al., and Pørksen et al.—were consistently rated as low risk across all domains. Conversely, smaller and short-term studies, such as those by Janiani and Ravindran, Yadav et al., Sakhare et al., and Hasslöf et al., demonstrated higher risk of bias, particularly due to limited sample size, unclear or unreported randomization procedures, and incomplete outcome data. The most frequent sources of bias were identified in D1 (randomization process) and D3 (missing outcome data), reflecting either insufficient detail in reporting allocation methods or attrition that could influence study validity. By contrast, D4 (measurement of outcomes) was consistently rated at low risk, as most studies employed objective microbiological or validated clinical measures (e.g., ICDAS criteria, PCR, standardized plaque scoring). Taken together, these findings indicate that while the overall evidence base is moderately robust, caution should be applied when interpreting results from smaller or short-duration RCTs, as these contribute greater uncertainty. The higher-quality, well-powered, multicenter RCTs provide stronger evidence to support the conclusions of this review (Figure 2).

## 4 Discussion

The present systematic review provides an updated synthesis of clinical evidence concerning the preventive role of probiotics against dental caries. By focusing exclusively on randomized controlled trials conducted during the last decade, this work aimed to overcome the heterogeneity and methodological limitations that characterized earlier reviews, providing a more consistent evaluation of clinical, microbiological, and immunological outcomes.

### 4.1 Microbiological effects of probiotics

The collective findings from the reviewed studies indicate that probiotics, particularly strains of *Lactobacillus* and *Bifidobacterium*, exhibit promising potential in reducing *Streptococcus mutans* (SM) levels and mitigating caries progression in pediatric populations. Despite variations in study design, intervention periods, and probiotic strains used, consistent trends emerge supporting their role in oral health.

Yadav et al. (2014) demonstrated a statistically significant decrease in S. mutans (*p* = 0.003) after consuming Lactobacillus casei Shirota, while Janiani and Ravindran (2022) found that both Yakult (*L. casei Shirota*) and enKor-D (*Lactobacillus reuteri and Bifidobacterium*) significantly reduced *S. mutans* counts, with enKor-D showing a more pronounced effect (66, 74). Similarly, Wattanarat et al. and Manmontri et al. reported a reduction in S. mutans along with an increase in salivary immune peptides, specifically human neutrophil peptides 1-3 (HNP1-3), suggesting an immune-modulatory role of probiotics in oral defense mechanisms (67, 68).

Javid et al. showed that probiotic yogurt containing *Bifidobacterium lactis Bb12* significantly reduced *S. mutans* and *Lactobacillus* levels in young people with early-stage caries (69). Ghasemi et al. compared probiotic yogurt with xylitol-containing chewing gum and reported significant bacterial reductions in both groups, with probiotic yogurt showing slightly greater efficacy (78). However, bacterial levels tended to rise again after cessation of the intervention, though remaining below baseline values, indicating that probiotic-induced microbial changes may be transient without continuous administration.

### 4.2 Clinical outcomes and long-term caries prevention

Studies assessing long-term caries progression provide compelling evidence for probiotics as preventive agents. Rodríguez et al. and Sandoval et al. evaluated *Lactobacillus rhamnosus SP1* over 10 months in high-caries-risk preschool children (64, 65). Both studies revealed a lower incidence of new cavitated lesions in probiotic groups, indicating a protective effect against caries development. Rodríguez et al. further quantified this effect, showing a 35% reduction in caries odds (OR = 0.35). Despite these promising results, heterogeneity among strains, dosages, and intervention duration complicates direct comparison across studies. Janiani and Ravindran highlighted differences between probiotic formulations, but the optimal strain, dose, and regimen for caries prevention remain uncertain (74). The transient nature of probiotic effects was also noted by Sakaryalı Uyar et al., where *S. mutans* levels significantly decreased after probiotic administration but gradually returned toward baseline values over time (73), suggesting that sustained or repeated probiotic use may be required to maintain benefits. Comparative studies such as Krupa et al. demonstrated that probiotics were more effective than xylitol in reducing *S. mutans* levels in children (77), reinforcing their potential as a non-invasive preventive strategy. However, trials by Pørksen et al. and Mughal did not show significant effects on plaque accumulation or gingival inflammation despite reductions in bacterial load (70, 71).

### 4.3 Immunological and host defense mechanisms

Beyond their microbiological actions, probiotics may exert immunomodulatory effects that enhance host resistance to caries. Wattanarat et al. and Manmontri et al. found that probiotic administration increased salivary concentrations of HNP1–3, key antimicrobial peptides in innate immunity (67, 68). These molecules help suppress cariogenic bacteria and maintain mucosal barrier integrity. One of the most notable findings was reported by Pørksen et al., where daily use of a lozenge containing *Lacticaseibacillus rhamnosus LGG*®, *L. paracasei L. CASEI 431®,* and 2% arginine significantly reduced caries increment (*p* = 0.007) (71, 80). The presence of arginine, a known immune-modulating prebiotic, supports the hypothesis that symbiotic formulations can combine antimicrobial and immunoregulatory effects, contributing to a more balanced and less cariogenic oral environment. However, the clinical relevance of these immunological effects remains uncertain. Further studies are needed to determine whether such immune activation directly translates into long-term caries reduction.

### 4.4 Specific interventions and pediatric applications

Several studies have explored the role of probiotic-enriched foods, such as curd and yogurt, in modifying the oral microbiota. Sakhare et al. and Duraisamy et al. both observed significant *S. mutans* reduction following probiotic curd consumption, with the latter suggesting greater efficacy for probiotic curd compared to synbiotic curd (75, 76). In pediatric populations, probiotics have shown potential in preventing early childhood caries (ECC). Villavicencio et al. observed reductions in salivary *S. mutans* and *Lactobacillus spp.* counts among preschool children consuming probiotic-enriched foods (81). Staszczyk et al. also reported decreased caries progression in high-risk pediatric cohorts following short-term Lactobacillus salivarius supplementation (82, 84). In orthodontic patients, Gizani et al. investigated *L. reuteri* supplementation for white spot lesion (WSL) prevention, finding reductions in *S. mutans* levels but no significant decrease in WSL incidence (79). These findings suggest that while probiotics may contribute to oral microbial balance, their clinical benefit may depend on patient-specific factors such as compliance and hygiene practices.

## 5 Strenght and limitations

### 5.1 Strengths

This systematic review provides an updated and focused synthesis of the evidence on the use of probiotics for dental caries prevention in children and young adults, addressing a clinically relevant population that has not been specifically analyzed in previous reviews. All included studies were randomized controlled trials (RCTs), and the risk of bias was assessed using the RoB 2 tool across its five domains, ensuring methodological rigor and reliability. The review integrates clinical, microbiological, and immunological outcomes, offering a comprehensive evaluation of probiotic efficacy. The comparative analysis of different strains, delivery vehicles, and administration protocols adds practical insight into factors influencing treatment effectiveness.

### 5.2 Limitations

However, several limitations must be acknowledged. The heterogeneity in probiotic strains, dosages, delivery methods, and follow-up periods limits comparability and prevents meta-analytical synthesis. Most studies assessed short-term interventions, providing limited evidence on long-term probiotic effects and colonization stability. The lack of standardized protocols further restricts the ability to define optimal regimens. Moreover, as the included trials primarily involved pediatric and young adult populations, the findings may not be generalizable to older individuals or those with systemic conditions. Future standardized, long-term RCTs are warranted to confirm efficacy, determine ideal administration parameters, and clarify underlying mechanisms in caries prevention.

## 6 Conclusions

Probiotics, especially *Lactobacillus* and *Bifidobacterium strains*, show promising potential in reducing *Streptococcus mutans* levels and preventing dental caries, particularly in children and high-risk groups. While short-term benefits have been consistently observed, the effects are often transient, suggesting the need for continuous use. Synbiotic combinations may enhance efficacy, but optimal strains, dosages, and regimens remain unclear. Overall, probiotics represent a safe and natural adjunct to caries prevention, but further standardized, long-term clinical trials are essential to confirm their effectiveness and guide clinical application.

## Data Availability

The original contributions presented in the study are included in the article/Supplementary Material, further inquiries can be directed to the corresponding author.
